# Stakeholder perspectives on adapting and disseminating Ghana’s physical activity guidelines: a qualitative study

**DOI:** 10.1186/s12889-021-12250-1

**Published:** 2021-12-11

**Authors:** Laura E. Balis, Kwame Kesse Adjei, Solomon Nyame, Jones Opoku Mensah, Kwaku Poku Asante

**Affiliations:** 1 Pacific Institute for Research and Evaluation, Louisville, KY USA; 2 University of Wyoming Extension, Lander, WY USA; 3 Kintampo Health Research Centre, Ghana Health Service, Kintampo, Ghana

**Keywords:** Physical activity guidelines, Ghana, Adaptation, Cultural appropriateness, Implementation, Dissemination

## Abstract

**Background:**

Ghana is facing the public health “double burden” of both communicable and chronic diseases. To combat increased chronic disease prevalence, physical activity promotion efforts are necessary. The Ministry of Health (MOH) developed physical activity guidelines in 2009, but community members are unaware of the guidelines and sample activities (e.g., ballroom dancing) are not culturally appropriate. The purposes of this study were to investigate 1) dissemination of the physical activity guidelines through MOH and Ghana Health Service (GHS) and 2) culturally appropriate physical activities.

**Methods:**

Data were collected in urban and rural areas of Ghana through focus groups (*N* = 2) with community representatives and in-depth interviews (*N* = 15) with GHS health workers. Focus group and interview questions included recommended types of physical activity; interview questions included dissemination factors based on Diffusion of Innovations. The research team analyzed the data through an inductive, grounded theory approach.

**Results:**

Together, the focus groups and in-depth interviews generated 942 meaning units coded into themes of Physical Activity Perceptions (*N* = 337 meaning units), Suggested Physical Activities (*N* = 317), and Dissemination and Implementation Factors (*N* = 290). Participants had positive perceptions of physical activity but expressed concerns over individual abilities; barriers included the built environment and a lack of time. Suggested physical activities included walking, jogging, football, and dancing for adults; traditional games and football for youth, and walking and daily chores for older adults. Participants noted that guideline implementation was influenced by leadership engagement at multiple levels, relative advantage, and compatibility. Respondents suggested implementation strategies to resolve barriers, including involving partner organizations and developing an implementation plan. Participants were largely unaware of the physical activity guidelines; typical dissemination methods included written materials and the internet.

**Conclusions:**

The results of this study suggest that physical activity guidelines should include familiar physical activities such as traditional games. Results also suggest that public health workers within GHS experience challenges in disseminating the physical activity guidelines. Adapting, disseminating, and implementing physical activity guidelines is a necessary step in increasing physical activity levels and preventing chronic diseases. These results contribute to understanding translation of physical activity policy to practice.

## Background

Ghana, as well as other developing countries across the world, is facing the public health “double burden” of both communicable and chronic diseases [1–4]. Chronic disease prevalence is on the rise as urbanization and sedentary work increase [5–8]. The benefits of physical activity as a modifiable risk factor in chronic disease prevention have been well established [9–11]. However, rates of physical activity in Ghana have decreased across the life span, with most Ghanaian youth not meeting physical activity recommendations [12, 13], and women, older adults, and those living in urban areas less active than their peers [5, 7, 8, 14, 15]. Physical activity promotion efforts are necessary to address the developing world’s new public health challenge and combat the rise in chronic disease [16, 17].

To respond to the need for physical activity promotion, Ghana’s Ministry of Health (MOH) developed physical activity guidelines in 2009 as part of the World Health Organization (WHO) Global Strategy on Diet, Physical Activity, and Health, written with a goal of assisting WHO member states with developing a national physical activity plan [6, 17]. As recommended by the WHO, the guidelines align with the 1996 Report of the US Surgeon General on Physical Activity and the National Institutes of Health Consensus Development Panel, and recommend that adults engage in 150 min of moderate-intensity aerobic activity (or 75 min of vigorous-intensity aerobic activity, or an equivalent combination of both) and two sessions of muscle-strengthening activities (targeting major muscle groups) per week, and children/adolescents engage in 1 h or more of moderate to vigorous intensity physical activity per day [6].

Establishing evidence-based national physical activity guidelines is a sound first step in developing implementation plans [18], communication strategies, and evidence-based interventions. However, across countries, there is a well-documented gap in translating research findings into improved public health, and moving physical activity policy to practice is no exception [19–24]. Dissemination strategies (i.e., targeted approaches to spread interventions to specific audiences) [25] are needed to package and communicate the guidelines to both public health practitioners and the public. If guidelines are not communicated to the public, they may not be aware of the benefits of physical activity, recommended types and amounts, and how to achieve them.

When policies do reach public health practitioners and community members, for messages to be relatable, the policies need be to culturally appropriate. Adapting health promotion interventions (programs, policies, or practices) for new settings is crucial to enhance cultural appropriateness [26–29]. Without images, language, examples, or other cultural features that are relevant, policies may fail to reach the target audience or be ineffective [30, 31]. The recommended process for scaling-out [32] interventions to new delivery systems and populations is: assessing priority population needs, determining necessary changes to the intervention, consulting experts, adapting, and then implementing and evaluating the adapted intervention [33].

Evidence suggests that Ghana’s physical activity guidelines have not been disseminated to end users, nor adapted for cultural appropriateness. A study on older Ghanaian adults’ perceptions of physical activity found that participants were not familiar with the MOH physical activity guidelines or how to meet them (e.g., what types of exercise counts as strength training or as moderate intensity aerobic activity) [34]. At the organizational level, no published research has been found on the implementation of physical activity programs or policies developed based on the guidelines in the 12 years since they have been published, which suggests a lack of dissemination to public health practitioners and policy makers.

Additionally, the MOH physical activity guidelines may not be culturally appropriate. As the MOH physical activity guidelines were adapted from United States-based recommendation, the guidelines include activities such as swimming and ballroom dancing as examples of moderate to vigorous level aerobic activity [6]. In previous research, older Ghanaian adults were queried about preferred types of physical activity, and reported that these are not activities they engage in (e.g., “we don’t know how to swim, we live here, but we don’t know how to swim”) [34]*.* Rather, the most common types of recommended aerobic physical activities among these older adults were walking, dancing, jumping/skipping rope, jogging, and team sports [34]. Literature on preferred types of physical activity in Ghana is sparse, with one other study reporting preferences for dancing, group classes at the gym, and walking among adult women in Accra, Ghana’s largest urban area [35]. Thus, there is a need to determine culturally appropriate physical activities across the life span throughout Ghana.

Taken together, to improve compliance with national physical activity guidelines, these guidelines need to be disseminated to target populations of delivery agents (to eventually be disseminated to end users) and adapted to include culturally appropriate physical activities as relevant examples. To understand why the MOH guidelines were not disseminated to end users and what example activities should be included, the purpose of this study was to investigate stakeholder perspectives of 1) dissemination of the physical activity guidelines through Ghana Health Service (the implementation arm of the MOH) and 2) culturally appropriate physical activities across the life span. For the purpose of this study, we draw upon implementation science literature on adaptation, and operationalize "culturally appropriate" as relevant to the priority population and local conditions [26–29].

## Methods

### Study design

The study employed a qualitative design implemented through a collaboration between the University of Wyoming and Kintampo Health Research Centre (KHRC), a research centre of Ghana Health Service (GHS). The Kintampo Health Research Centre Institutional Review Board approved this research protocol. All participants provided informed consent via a written consent form. Data were collected in 2019 and analyzed in 2020.

### Study setting

The study was conducted within the Ghana Health Service. GHS is an autonomous agency under the MOH. It is responsible for implementing national policies and ensuring access to quality services. The GHS has a national headquarters located in the Greater Accra Region in addition to 16 regional health directorates. The various regional health management teams have municipal and district health management teams under their jurisdictions. The regional health directorates in the Upper West and Bono East as well as the national headquarters in the Greater Accra region were used for this study.

### Data collection

The research team collected data through 1) focus groups with Community Health Management Committees, who served as community representatives and 2) in-person interviews with GHS health workers. Community Health Management Committees are groups designed to give guidance to GHS on health delivery services at the community level. Members are nominated by the community to represent them in health related issues [36]. The research team developed semi-structured guides for both the focus groups and interviews.

#### Community Health Management Committee focus groups

Questions included preferred types of physical activity and facilitators/barriers to physical activity by age group (youth, adults, and older adults) as well as awareness and perceptions of the MOH physical activity guidelines. Participants also completed a brief survey consisting of demographic items. Focus groups lasted approximately 60 min, and community leader participants were compensated for their time with a small gift (refreshments and a personal care item).

#### GHS health worker in-depth interviews

The GHS health worker interviews were designed to understand from staff perspectives why the MOH physical activity guidelines have not been more widely disseminated and what culturally appropriate physical activities should be included. Questions were based on the Diffusions of Innovations Theory, including aspects of the physical activity guidelines (compatibility, complexity, trialability, and observability), communication channels (how ideas are transmitted from one person to another), and the social system (the individuals who adopt the innovation) [37], as well as similar research on physical activity plan dissemination [18]. Questions also included standard demographic items and questions on preferred types of physical activity and facilitators/barriers. Interviews lasted approximately 45 min. Participants received monetary reimbursement to cover their time and transportation to and from the interview venue.

### Participants and recruitment

Focus group were conducted with Community Health Management Committees members made up of community leaders, household heads, church leaders (e.g., pastors and imams), chiefs, and linguists. Committee members were purposively sampled by GHS municipal/district-level health workers and contacted through letters with an invitation to participate in the focus groups. For the interviews, eligible participants were health workers employed by GHS who were responsible for disseminating public health guidelines (e.g., directors, program managers, program coordinators, public health nurses, and nutrition officers). The health workers were purposively sampled at the national, regional, and municipal/district levels and were contacted by official letters from KHRC with invitations to participate in the in-depth interviews.

### Analysis

Focus groups and in-depth interviews were audio-recorded. Focus group were conducted primarily in Twi (Ghanaians’ local language), while in-depth interviews were conducted in English. Focus group recordings were transcribed into English in Microsoft Word by bilingual Ghanaian authors.

Fifteen health workers were recruited for in-depth interviews. The research team gave respondents the choice of participating in the interview or providing a written response to the interview prompts. The majority (*n* = 11) opted to answer in writing. These written responses were complied with the in-depth interview and focus group transcripts. This method (i.e., collating multiple data sources) allowed us to refine our approach to respond to contextual factors and prioritize the community-engaged essence of this work [38, 39].

The research team analyzed the data through an inductive, grounded theory approach [40]. Transcripts and written responses were independently coded by authors to identify meaning units (words, phrases, or sentences containing related content conveying a specific thought or idea) [41]. After identifying and categorizing meaning units, the authors performed sorting and collapsing of similar meaning units. Authors reviewed the other authors’ meaning units and analysis. Audit trails [42] were preserved for all data, including audio recordings, transcripts, and all coding documents.

## Results

### Demographics

Health worker interviewees (*n* = 15) had a mean (±SD) age of 38.9 (±5.93) years, mean (±SD) years of GHS employment of 6.1 (±4.45) years, were primarily female (53%) and Christian (73%), and had earned a bachelor’s degree or higher (67%). See Table 1. Focus group participants (*n* = 17) had a mean (±SD) age of 40.1 (±10.13) years, were Christian (100%) and primarily male (82%), and had not completed a university degree (100%).

**Table 1:** Demographic variables of Ghana health service interview participants (*N* = 15) and community stakeholder focus group participants (*N* = 17)

Demographic variables	Interview Participants (***N*** = 15)	Focus Group Participants (***N*** = 17)
	**Mean (**±**SD)**	
**Age**	38.9 (±5.93) (*n* = 10)	40.1 (±10.13)
**Years in Job**	6.1 (±4.45) (*n* = 11)	Not applicable
	**N (%)**	
**Gender**		
Female	8 (53)	3 (18)
Male	3 (20)	14 (82)
Not reported	4 (27)	0 (0)
**Highest education level**		
Masters’ Degree	3 (20)	0 (0)
Tertiary / University Degree	7 (47)	0 (0)
Secondary / Senior High School	1 (7)	3 (18)
Junior High School	0 (0)	8 (47)
Primary School	0 (0)	6 (35)
Not reported	4 (27)	0 (0)
**Religion**		
Christian	11 (73)	100 (100)
Muslim	1 (7)	0 (0)
Not reported	3 (20)	0 (0)
**Region**		
Bono East	6 (40)	17 (100)
Upper East	5 (33)	0 (0)
Greater Accra	4 (27)	0 (0)

### Qualitative

The focus groups (*N* = 2) and in-depth interviews (*N* = 15) generated 942 meaning units; they were coded into themes of Physical Activity Perceptions (*N* = 337 meaning units), Suggested Physical Activities (*N* = 315 meaning units), and Dissemination and Implementation Factors (*N* = 290 meaning units). Meaning units around Physical Activity Perceptions were divided into subthemes of A) Barriers to Physical Activity, B) Personal Beliefs, and C) Facilitators to Physical Activity. See Table 2. Barriers to physical activity included multiple challenges related to the built environment, particularly related to urbanization. Participants reported that “For me we achieve optimum exercise through sports but there are no spaces in the cities; the chiefs and governments are selling our football pitches [health worker],” and “I think our way forward is creating enabling environment for diet and physical activity. If there are no enabling environments; we will only come out with beautiful ideas but it can never be implemented even when people are taught, it will be very difficult for them to practice because there are no enabling environments [health worker].” Another barrier reported was a lack of time, especially related to school and work commitments: “Straight from the school, they drive home and do their homework and the next thing it is evening [health worker].”

**Table 2:** Qualitative results for theme: physical activity perceptions (*N* = 337 meaning units)

Subtheme (*N* = meaning units)	Category (*N* = meaning units)	Example meaning unit
Barriers to physical activity (*N* = 232)	Built environment (*N* = 51)	If you come out with this document, it will be a shelve documents; the facilities are not available.
	Lack of time (*N* = 41)	… you will have to be at work by 8 AM and so you will have to get up early to do some jogging and in the evening from close of work the traffic in Accra; what time will you get to walk and do all kind of things as such. There are hardly for you to get space during the weekdays because of these activities and the weekends are always packed with activities.
	Parenting (*N* = 24)	I think when you do not want the child to be hurt; when here there are restrictions from parents or guidance. You may think the child might get hurt through exercise so you prevent the child from doing it.
	Ill health (*N* = 23)	Some are also as a result of sickness. Sickness could prevent one from doing exercise.
	Lack of education (*N* = 20)	Sometimes the knowledge is also a factor, if I am not aware of how importance PA is, when there is even time I will rather prefer sitting and chatting rather than going out for a walk.
	Low motivation (*N* = 16)	Lack of motivation to exercise [prevents regular physical activity].
	Injury concerns (*N* = 14)	Because at their age [elderly] that is when weak bones set it, they become susceptible to fractures.
	Gender roles (*N* = 12)	If the males are not around [the females can pound the fufu].
	Lack of support (*N* = 9)	No encouragement from friends and family [prevents regular physical activity].
	Sedentary hobbies (*N* = 9)	And also gadgets like televisions, phones, television games and computer games; is sort of engages them so they are not able to go out and play.
	Substance abuse (*N* = 7)	Alcoholism [prevents adults from regular physical activity].
	Poor nutrition (*N* = 6)	Hunger is also a factor. When they are satisfied they can play a lot. Like most children, when they are hungry, they will always be in the house but immediately they get satisfied, they move out to play.
Personal beliefs (*N* = 58)	Individual abilities (*N* = 36)	Some is also due to lack of strength. If you are not strong you cannot exercise.
	Benefits of PA (*N* = 8)	The little I will like to add to encourage others is that, when you exercise it makes you live long so we should try am much as possible to be able to do it.
	Preferred day/time (*N* = 8)	Exercises at week ends.
	Meaning of PA (*N* = 6)	Exercise can be translated into Twi as “apomutenene” (stretching of muscles).
Facilitators to PA (*N* = 47)	Education (*N* = 25)	When we let people know, it is always good to have some exercise within the week or for some minutes, that will encourage individuals or making them know the benefits, what they can actually gain from exercise, it will encourage them.
	Facilities/equipment (*N* = 9)	With the elderly, I think the club/gym level is a better choice.
	Social support (*N* = 6)	If they see their colleagues engaged in athletics it encourages them to also participate in it. They then call each other when they are going to play.
	Good nutrition (*N* = 4)	What will help them [young ones] is to eat carbohydrate rich foods such as cassava, maize. When they eat very well they can exercise very well.
	Group exercise (*N* = 3)	Group exercise such as pension groups.

Participants shared beliefs around individual abilities to engage in physical activity. For example, multiple concerns were expressed around older adults’ abilities: “Age is also a factor; every age has a kind of exercise they can indulge in [community representative].” and “You cannot as an old man go jogging; that will add to their problems rather than solving them [health worker].” Participants also expressed perceived benefits of physical activity, e.g., “Also, high blood pressure, exercise avoids high blood pressure [community representative].” Preferred days and times for physical activity were shared, especially related to being active on weekends when more free time is available.

Regarding facilitators, respondents felt that education was key to increasing physical activity levels: “Education is also important. Some do not know the importance of exercise so when he is being educated on the importance of exercise and he get to understand it they will be ready to do it [community representative].” Facilities and equipment were also perceived as important to facilitating physical activity. In particular, several participants mentioned a need for gyms for older adults to exercise safely, e.g., “They (the elderly) need the gym most, where their exercises can be regulated to suit their age [health worker].”

Suggested Physical Activities were divided into subthemes of A) Adults, B) Youth, and C) Older Adults. See Table 3. Participants suggested that adults should engage in walking (“but we should consider doing some walking [health worker]”), jogging, football, dancing, and other aerobic activities. For youth, respondents mentioned traditional games (“They are good fun filled activities that can also provide some health benefits. I know the ampe [health worker]”), football, and other aerobic activities. As for older adults, the most commonly suggested activities were walking and daily chores, e.g., “My mum like the gardening most [health worker].”

**Table 3:** Qualitative results for theme: suggested physical activities (*N* = 315 meaning units)

Adults (*N* = 196)	Walking (*N* = 28)	Even if I am walking, it is an exercise.
	Jogging (*N* = 26)	Exercise is running from one point to the other.
	Football (*N* = 18)	In this town, the exercise they do is playing football.
	Dancing (*N* = 13)	Yeah. You can dance anywhere.
	Other aerobic activities (*N* = 14)	… beach boxing, that is what those at the beach likes to do.
	Biking (*N* = 11)	Riding of bicycle [is recommended for adults].
	Skipping (*N* = 10)	And then skipping rope.
	Swimming (*N* = 10)	Swimming in a river.
	Basketball (*N* = 8)	Basketball [is recommended for adults].
	Daily chores (*N* = 7)	And sweeping is an exercise.
	Volleyball (*N* = 7)	Volleyball [is recommended for adults].
	Weeding (*N* = 7)	Weeding is an exercise.
	Athletics (*N* = 5)	Athletics [are recommended for young adults].
	Body weight exercise (*N* = 6)	Okay we also have push-ups.
	Traditional games (*N* = 6)	There is also “awantwo”
	Table tennis (*N* = 5)	You play table tennis.
	Weightlifting (*N* = 4)	Lifting of metals in order to gain strength.
	Tennis (*N* = 3)	Then you should add long tennis as well.
	Jumping (*N* = 3)	… as well as some kind of jumping for some hours.
	Handball (*N* = 3)	Handball [is recommended for young adults].
	Stretching (*N* = 2)	Exercise means stretching up your joint.
Youth (*N* = 71)	Traditional games (*N* = 16)	So, we should try and go back to our root. With these ball room dancing, I don’t know what it is.
	Football (*N* = 12)	And they are always happy when in such activities like football and this could help them to do exercise.
	Other aerobic activities (*N* = 10)	Tennis, etc. [is recommended for children].
	Games (*N* = 8)	With the children, I will recommend them playing some hide and seek among themselves.
	Jogging (*N* = 6)	Children can run.
	Playing (*N* = 4)	For children I think we should just let them play, run around.
	Skipping (*N* = 4)	Skipping rope is also an exercise.
	Dancing (*N* = 3)	Dancing [is recommended for children].
	Jumping (*N* = 3)	They [children] can jump.
	Swimming (*N* = 3)	Is a nice activity, it makes you feel tired as you flop your hand and legs, by the time you finish you will fee pains in your hands and legs joints. Swimming is a nice thing but if you haven’t done some before you will find it difficult.
	Daily chores (*N* = 2)	I want to add what the youth can do; they can also pound fufu.
Older adults (*N* = 48)	Walking (*N* = 20)	I think the elderly should be doing more of brisk walking, walking.
	Daily chores (*N* = 10)	Ironing [is recommended for the elderly].
	Weeding (*N* = 4)	They can also weed.
	Dancing (*N* = 3)	Dancing [is recommended for the elderly].
	Other aerobic activities (*N* = 3)	Aerobics [are recommended for the elderly].
	Biking (*N* = 3)	Whenever they meet, the exercise they should be doing is riding of bicycle.
	Stretching (*N* = 3)	And stretching to some extent like stretching all parts of the body, I think that will be very good for them [the elderly].
	Traditional games (*N* = 2)	Or the “chaskele” [is recommended for the elderly].

Dissemination and Implementation Factors shared by health workers were divided into subthemes of A) Contextual Factors, B) Implementation Strategies and Suggestions, C) Communication Sources and Channels, and D) Innovation-Decision Process. See Table 4. Contextual Factors included leadership engagement, relative advantage, compatibility, executing, knowledge and beliefs, and relative priority. Leadership engagement was most commonly mentioned, with respondents sharing several levels of staff who should be involved with the physical activity guidelines. This included directors, nutrition officers, health promotion officers, and physical educators: “Health Promotion officer, etc. These caliber of staff can facilitate the implementation of the guidelines.” Respondents conveyed that implementing the physical activity guidelines presented a relative advantage over alternate solutions, as there was a lack of comparable guidelines available. They shared that the physical activity guidelines are compatible with existing programs and policies: “It would complement Regenerative Health which is an ongoing program. Regenerative Health talks of physical exercise as medicine.”

**Table 4:** Qualitative results for theme: dissemination and implementation factors (*N* = 290)

Contextual factors (*N* = 128)	Leadership engagement (*N* = 26)	Guidelines of such nature should be made available to the critical staff, e.g. NCD [non-communicable disease] coordinator, Nutrition Officer.
	Relative advantage (*N* = 26) Alternative guidelines not available (*N* = 20) Alternative guidelines available (*N* = 6)	I do not know anything of such [plans of physical activity in the region].
	Compatibility (*N* = 24) Compatible with policies and programs (*N* = 19) Unsure (*N* = 5)	There may be no complications. The guidelines will and can be easily integrated with work at the GHS.
	Executing (*N* = 17) Agency disconnect (*N* = 6) Agency role (*N* = 5) Funding (*N* = 4) Agency structure (*N* = 2)	But we have not been engaged at the implementation level and that is the disconnect concerning the dietary and physical activity guidelines.
	Knowledge and beliefs (*N* = 16)	Thus [exercise/physical activity is] very important and should be a priority to all.
	Relative priority (*N* = 8)	But because initially we did not have over-nutrition as a problem in Ghana so we were not really addressing it but now is coming up, all surveys and researches are pointing out that over-nutrition, diabetes, hypertension and all other diet related conditions are now becoming a big problem so there is an urgent need for GHS to come out with a program and a document like this will be a backbone for such a particular problem. So as we move forward this should be a way we should go. Currently I think there is an urgent need for that because we have heard on the double burden of malnutrition in Ghana, i.e. over nutrition and undernutrition
	Engaging (*N* = 6)	But if you want a place where the implementation will take place then it will be at the community level
	Planning (*N* = 3)	Yes. In developing the guidelines, we were part of it; all relevant stakeholders or technocrat within the nutrition fraternity, including academia, research and relevant international or donor agencies and the Ghana Health Service came together to put these documents together.
	Reflecting and evaluating (*N* = 2)	This guideline was developed in 2009 and it is about 10 years old now; new issues are emerging in public health and I think the document need to be reviewed and updated with current trends; obviously, there are new strategies, the situation 10 years ago is not the same today; looking at rates of obesity and disease prevalence, there have been changes.
Implementation strategies and suggestions (*N* = 64)	Involve partner organizations (*N* = 33) Education (*N* = 24) Employment (*N* = 6) Health care (*N* = 3)	All schools should make physical activities compulsory in a well-structured form which will not compete with academics.
	Implementation plan (*N* = 11)	When implementing a program, you should have an implementation framework. When you formulate a documents as such, you should have another document which will have the plan on how it is going to be disseminated and utilized and everything should be clearly spilled out; how is its implementation going to be monitored, at what point do you need to re-visit the strategy and do a review on it and the stakeholders who have come together to formulate this documents, how are they going to implement it in their various activities.
	Train stakeholders (*N* = 6)	Stakeholders will have to be trained.
	Change policies (*N* = 4)	All these things [built environment, parks, physical activity breaks] are policy issues that need to be addressed.
	Hire staff (*N* = 4)	These should be personnel appointed [to see to it that is implemented].
	Create unit (*N* = 2)	There would have been a unit of such; a team that will reflect the essence of the program and they will go through as the normal program; is not going to be a project but a program which is more sustainable.
	Capacity building (*N* = 2)	People capacity should be built on how to use the guidelines; I do not know if that was done but I cannot speak to that because I am not in the lead agency but all I know was I was not part of any capacity building team.
	Resources (*N* = 2)	Strong advocacy for more resources to be allocated to those programmes.
Communication channels and sources (*N* = 50)	Written materials (*N* = 16)	I think pamphlets will do [to incorporate physical activity guidelines into the health system].
	Internet (*N* = 11)	Any physical activity I talk [about] or recommend to people are materials I read online.
	Colleague (*N* = 9)	It [physical activity guidelines] was an information I heard from a friend. He said is some kind of exercise activities.
	Training (*N* = 8)	Through dissemination meetings, when such documents are developed there is usually dissemination meeting where stakeholders who will be working with the documents are called to share with them what it entails and the new directions.
	Email (*N* = 3)	Emails [are a common way to learn about new guidelines or policies].
	Media (*N* = 3)	Seeing it on the various WhatsApps platforms.
Innovation-decision process (*N* = 48)	Knowledge (*N* = 40) Unaware of guidelines (*N* = 29) Aware of guidelines (*N* = 11)	What I want to add is that we those who have gathered here, you asked us whether we know/have heard about the guidelines and we said we have not.
	Implementation (*N* = 8) Have not used guidelines (*N* = 7) Have used guidelines (*N* = 1)	Though I have never used the guideline.

Implementation strategies and suggestions mentioned by health workers included the perception that partner organizations should be involved in implementing the physical activity guidelines. These partners included education, health care, and employment sectors: “Incorporating physical activities in the Ghana Education Service school curriculum,” “It can be integrated into our existing clinic sites e.g. diabetes clinics, child welfare clinics, and family planning corners,” and “For working groups, employers should embark on routine activities.” Health workers also noted that there was a need for an implementation plan to execute the guidelines: “and most importantly, there should be an implementation plan for the dietary and physical activity.”

Considering communication sources and channels for dissemination, participants primarily learn of new innovations through written materials (e.g., “letters,” “internal memos,” and “posters”) and the internet (“Now, we use both, the service has a website, so when we are done with the documents, we post it there.”) Finally, concerning the innovation-decision process, health workers shared that they did not have knowledge of the physical activity guidelines (“I really have no idea about such guidelines”) and had not implemented them (“At the implementation level, we those under Ghana Health Service, [blinded] Department have not done anything with dietary and physical activity since its implementation.”)

## Discussion

This study investigated stakeholder perceptions of physical activity guidelines dissemination and culturally appropriate physical activities. The participants suggested physical activities and explained barriers, facilitators, and personal beliefs around physical activity. Additionally, the health workers interviewed shared meaning units related not only to challenges disseminating the physical activity guidelines, but also to implementing them. These results shared here can be useful in developing future versions of physical activity guidelines in Ghana along with dissemination and implementation strategies.

Participants primarily suggested aerobic activities for inclusion in the guidelines. These included walking or jogging, football, and dancing, which have been reported as common activities for Ghanaian adults and older adults in other research [34, 35]. Interestingly, although participants in other studies shared that they do not swim [34], swimming was suggested as an activity for adults and children, although it was also mentioned that the built environment does not include access to swimming facilities: “For swimming, because we do not have the facilities, it is always not available in most areas” and “but we cannot compare ourselves to those who have the swimming pool and other swimming infrastructure.” Participants also mentioned bicycling as a suggested activity and mentioned that there is a need for bicycles (“They will need bicycle. The people in my hometown need bicycle to do exercise”) and bicycle infrastructure (“bicycle trails close to home or workplace.”). While improving the built environment to facilitate more physical activities could increase access and physical activity levels, including example activities in the physical activity guidelines that most Ghanaians currently have access to may be a better strategy for increasing activity levels.

For example, traditional games were suggested as appropriate physical activities across the life span. These included ampe (a jumping game), antowankyire (a circular chase game), chaskele (a bat and ball game), and pilolo (a hide and seek game) [43, 44]. As participants mentioned the perceived healthfulness and positive perceptions of traditional games (“So, I think our local ones [traditional games] will be too healthful though I can’t mention all, but what we were doing about 40 years ago, I think we should go back to it.”), they are likely to have high acceptability and appropriateness [45] among community members. Related, there is recent interest in ampe both as a competitive sport as well as an obesity prevention intervention, with a recent study finding that a four-week ampe exercise program had positive effects on youths' body weight, blood pressure, and heart rate [43]. Including familiar, traditional activities in the guidelines (rather than unfamiliar activities common in other countries) could improve understanding and uptake.

While the focus group and interview respondents primarily discussed aerobic physical activities, they also mentioned two strength training activities – body weight exercise and weight lifting – for adults, but not for older adults. Daily chores were suggested across age groups (e.g., “scrubbing,” “cleaning their room”), as was stretching for older adults. These suggestions of light-intensity physical activities (rather than moderate to vigorous-intensity activities) and a focus on aerobic rather than strength training are similar to other research findings [34, 35]. More efforts are needed to provide education and clarification on types of physical activity and intensity levels to achieve disease-preventing benefits. As well, based on the activities suggested by participants, it may be useful to discuss sample physical activities in terms of domains, as described in the WHO’s Global Action Plan on Physical Activity [46]. These domains include work (including domestic work), travel (walking and cycling), and recreation (including sports), which can be combined to meet physical activity guidelines [46].

Concerning physical activity perceptions, participants believed in the benefits of physical activity, but had concerns about individual abilities that may limit physical activity participation. There were also concerns about injuries due to exercise – from youth through older adults. In addition, many of the suggested physical activities were described as “for girls” or “for boys” (e.g., “[I recommend football] mainly for boys.”); these gender perceptions may limit the physical activities perceived as available – especially for females. More work is needed to understand cultural factors and ensure that future physical activity policies clarify the appropriateness of physical activity across the life span, for all genders, and including all domains. This aligns with the WHO’s Global Action Plan on Physical Activity’s recommendation to improve access to physical activities “that are culturally appropriate and for people of all ages and abilities” [46].

Finally, lack of time was viewed as a barrier to physical activity in both adults and youth, with work and school schedules both preventing regular physical activity. Future versions of the physical activity guidelines could include partnering with school and employment sectors to implement physical activity policies, as was suggested by some respondents: … “sometimes when you go for workshops there are motility break where you can walk around.” These policies could pair well with improvements in the built environment to facilitate physical activity as a form of active transportation (to and from work or school) as well as increase the availability of recreational facilities (e.g., football pitches) for recreation during leisure time.

Regarding dissemination, the interviewees were largely unaware of the physical activity guidelines. Those who were familiar with the guidelines shared that they had not used them and relayed difficulties in using them. However, respondents believed that the physical activity guidelines would be compatible with current programs and policies. As for dissemination sources, respondents typically learned of new interventions from a variety of written materials. Packaging the physical activity guidelines into user-friendly, accessible publications [47] - disseminated online and through standard trainings - should be considered. Lastly, participants noted that the physical activity guidelines were developed in 2009 and would likely need to be updated prior to future implementation and dissemination efforts.

While the goal of the study was to understand dissemination of the physical activity guidelines (i.e., the targeted spread of information about an interventions), health worker participants also shared their perceptions of factors and strategies related to implementation (i.e., putting an intervention to use) [23, 25]. Leadership engagement and stakeholders at multiple levels – from community members and volunteers to regional and district directors – were considered important for disseminating and implementing new policies, and could be targeted for future dissemination and implementation interventions. Participants shared that other interventions may receive higher relative priority than physical activity promotion. This “double burden” of addressing both communicable and chronic diseases may result in government resources allocated to communicable disease prevention and management rather than physical activity promotion, as other researchers have noted that health care expenditure in Ghana disproportionately prioritizes communicable diseases [4, 48]. Related, some of the health workers also shared that their focus was on nutrition education rather than physical activity. Nutrition education may be a more natural focus area than physical activity promotion, as nutrition educators were already in place to address undernutrition and could shift efforts to overnutrition [48]. This focus area could be leveraged by better integrating physical activity promotion, as suggested in the WHO Global Strategy [17]. Finally, respondents suggested implementation strategies including creating an implementation plan and involving partner organizations.

The dissemination and implementation challenges discussed here are not uncommon to national physical activity guidelines [49–52]. Across multiple countries, PA guidelines typically lack implementation strategies; dissemination strategies are more often included, but are not usually evaluated [50, 52]. With the lack of strategic dissemination efforts, passive diffusion tends to occur, resulting in incomplete and inaccurate conveyance of national guidelines [53]. The most effective dissemination and implementation strategies are unknown [49, 50], and greater efforts are needed to identify, adapt, and test strategies to build the evidence on effective strategies in community (vs. clinical) settings (Harden SM, Balis LE, Houghtaling B, Powell B: Implementation strategies in community settings: an introduction to the Expert Recommendations for Implementing Change (ERIC) Compilation, submitted) and improve the overall public health impact of national physical activity guidelines.

### Limitations

While efforts were made to understand perceptions of stakeholders in diverse regions of Ghana, both focus groups were held in Bono East Region. Thus, we may have missed perspectives of community leaders in other regions. In addition, the Community Health Management Committees are designed to advocate for their communities and thus were used to represent community members’ perspectives. However, we did not engage the community members themselves, and may have received a narrower range of responses from the health management committee members than from diverse community members. For example, most committee members were men; female community members may have shared different perspectives. Including diverse perspectives will be important to decide what activities are considered “appropriate” (e.g., for girls, or in shared use spaces) and should be included in the guidelines. As well, we did not capture perspectives of other stakeholders who are involved with physical activity promotion, such as employees of Ghana Education Service and National Sports Authority. Finally, other physical activity guidelines in Ghana exist (e.g., The Physical Education and Sports implementation guideline and the National Education Curriculum), with documented challenges in implementation [12]. For this study, we chose to focus on the overarching MOH guidelines for physical activity across the life span rather than guidelines for specific populations and sectors.

## Conclusions

The goals of this study were to investigate culturally appropriate physical activities and dissemination of Ghana’s physical activity guidelines. Results suggest that guidelines should include familiar activities such as traditional games and clarify the appropriateness of physical activity across the life span and for all genders. The results also suggest that public health workers within GHS experiences challenges in disseminating the physical activity guidelines. Overall, a policy to practice gap remains, with the public largely unaware of and failing to meet guidelines [51]. Adapting and disseminating physical activity guidelines – and eventually, implementing programs based on these guidelines - is a necessary step in increasing physical activity levels and preventing chronic diseases.

## Data Availability

The datasets generated and analyzed during the current study are available from the corresponding author on reasonable request.

## Abbreviations

MOH: Ministry of Health
GHS: Ghana Health Service
WHO: World Health Organization
NCD: Non-Communicable Disease
